# Hard Palate Perforation in an Elderly Man With Dementia

**DOI:** 10.7759/cureus.15872

**Published:** 2021-06-23

**Authors:** Mohsin S Mughal, Ikwinder Preet Kaur, Hafsa Akbar, Syed H Abbas, Priya Angi

**Affiliations:** 1 Internal Medicine, Monmouth Medical Center, Long Branch, USA; 2 Department of Internal Medicine, Abington Jefferson Hospital, Abington, USA; 3 Pathology, Saint Barnabas Medical Center, Livingston, USA; 4 Pathology, Monmouth Medical Center, Long Branch, USA; 5 Geriatrics, Monmouth Medical Center, Leon Hess Cancer Center, Long Branch, USA

**Keywords:** hard palate, squamous cells carcinoma, elderly population, alzheimer’s dementia, unintentional weight loss

## Abstract

A 74-year-old male with a history of mild cognitive impairment presented to the emergency department with failure to thrive and generalized weakness. He was having difficulty swallowing leading to 30 pounds of unintentional weight loss in the last three months. His social history was significant for 12.5 pack-year smoking and drinking (two to three glasses of wine/day). The oral cavity examination revealed a large (3 × 2 cm^2^) defect with the erythematous border that encompassed the mid-palatal structures and emanated from the hard palate into his nasal cavity. Auto-immune work-up was negative. Palatal biopsy showed squamous cell carcinoma (SCC; well-differentiated). A diagnosis of locally advanced (stage IVa) oral cavity squamous cell carcinoma (OSCC) was made based on PET scan findings. A palatal obturator (prosthesis) was placed to improve his eating, prevent regurgitation. The patient opted for palliative care and did not want to pursue further treatment. He was discharged home with a regular follow-up visit.

## Introduction

Palatal perforation is a rare condition due to chronic necrosis of the bone which potentially leads to communication between oral and nasal cavities. Many disease entities can cause a clinically indistinguishable palatal perforation which can pose a difficult diagnostic dilemma. Acquired perforations can be seen with infections (syphilis), autoimmune diseases, drug abuse (cocaine), malignancies, and trauma [1]. Malignant causes of palate perforation include lymphoma, melanoma, squamous cell carcinoma (SCC), and acute lymphoblastic leukemia. In the oral cavity malignancies, the hard palate is one of the two least (1.3-5%) commonly involved sites, in addition to the retromolar trigone. Oral squamous cell carcinoma (OSCC) is relatively rare among hard palate tumors and constitutes only 12.1% of all neoplasms. Human papillomavirus (HPV) is an independent and most significant causal factor of OSCC with the HPV-16 genotype as the commonest culprit. OSCC mostly begins in the mucosa of aerodigestive tracts [2]. In HPV positive OSCC, the risk of palate involvement is documented as 0-33.3% in various review articles. The median age is 57 years with more male preponderance. OSCC usually starts as an oropharyngeal ulcer associated with dysphagia, mouth pain, weight loss, loose denture, and may progress to palate destruction leading to oronasal fistula in severe forms [3]. HPV-positive cancers have a better prognosis than HPV-negative tumors. Smoking and alcohol abuse significantly impairs the prognosis. Diagnosis is performed by histopathological examination and immunohistochemistry. Imaging studies like CT scan or MRI scan is performed to evaluate the tumor extent. The treatment options vary depending on the disease extent, a composite resection of the neoplasm with the palatal bone is commonly performed [4]. A multidisciplinary approach is necessary in case of perforation to prevent malnutrition, aspiration, or impaired speech. Small defects (class 1) defined as central defects that do not extend laterally to involve a tooth-bearing alveolar ridge can be managed with an obturator prosthesis. An alternative is a surgical reconstruction using a soft tissue flap. We have presented a case of hard palate perforation that proved to be malignant/OSCC in origin. Complete evaluation of palatal lesions is necessary including focussed history, physical examination followed by imaging, and tissue biopsy.

## Case presentation

**Figure 1:**
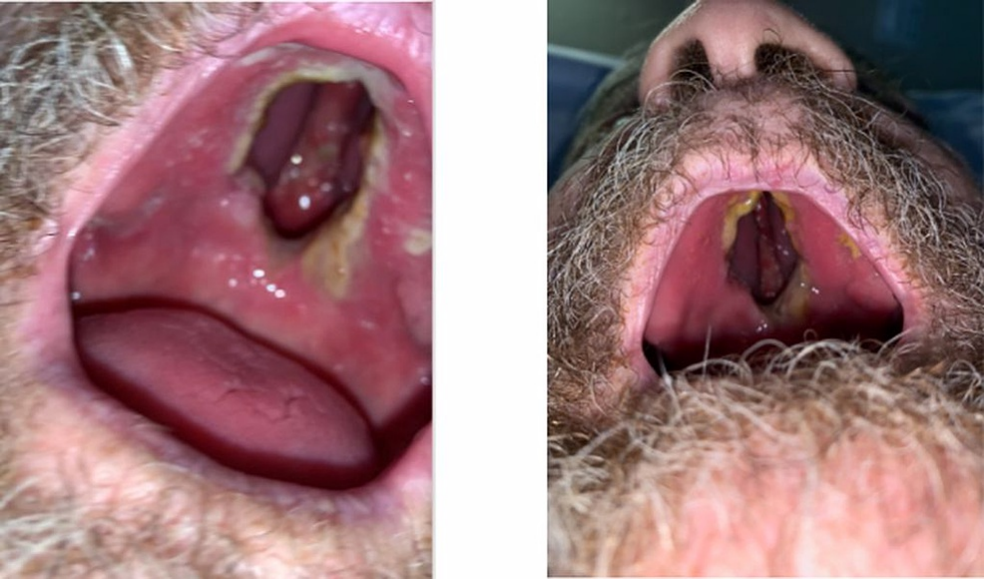
A 3 cm × 2 cm defect with an erythematous border that encompassed the mid-palatal structures and emanated from the hard palate into his nasal cavity.

A 74-year-old male with a history of mild cognitive impairment presented to the emergency department with failure to thrive and generalized weakness of three weeks duration. He was having difficulty swallowing which he related to a poorly fitted denture. He had thirty pounds of unintentional weight loss in the last three months due to loss of appetite. His past medical history was significant for diabetes mellitus type II, hypertension, major depressive disorder, chronic pain syndrome, and hepatitis C. His social history was significant for 12.5 pack-year smoking and drinking (two to three glasses of wine/day). He denied any utilization of recreational drugs. In the emergency department, his vital signs were, BP-104/59 mmHg, HR-97 bpm, respiratory rate-14/minute, temperature-97.4 °F, and oxygen saturation was 99% on room air. He weighed 40 kg, and his BMI was 13.84 kg/m^2^. On physical examination, he was alert, oriented, and appeared weak and malnourished. The ill-fitted complete maxillary denture was removed. The oral cavity examination revealed a large (3 × 2 cm^2^) defect with an erythematous border that encompassed the mid-palatal structures and emanated from the hard palate into his nasal cavity (Figure 1).

**Figure 2:**
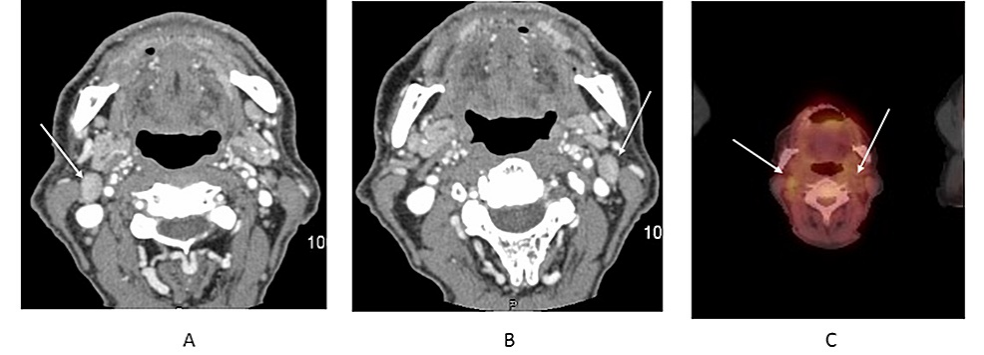
Contrast-enhanced CT of the neck demonstrates enlarged level lymph nodes bilaterally (A) and (B), corresponding to increased FDG avidity on PET CT scan (C). FDG: fluorodeoxyglucose.

There was no discrete mass or ulcer and mucosa surrounding this defect appeared normal. On palpation, the lesion was non-tender without any palpable lymph node on the head and neck examination. His lungs, heart, neurological, and abdominal examinations were unremarkable. Laboratory data at admission showed Hb-11.7 g/dl, WBC’s-9.6 K/CMM, and platelets-190 K/CMM. The chemistry panel (BMP) was within the normal range. AST-23 U/L, ALT-25 U/L, and serum alkaline phosphatase were 114 U/L. Auto-immune work-up including anti-dsDNA, anti-proteinase-3 antibodies, myeloperoxidase (MPO) antibodies were negative. The patient was managed conservatively, with nutritional supplementation and supportive care. Contrast-enhanced CT of the neck demonstrates enlarged level 2A lymph nodes bilaterally (Figure 2A and 2B), corresponding to increased fluorodeoxyglucose (FDG) avidity on PET CT (Figure 2C).

**Figure 3:**
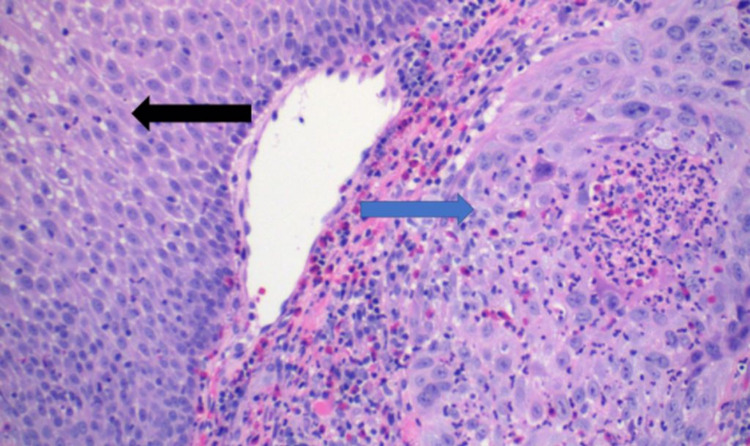
Hematoxylin and eosin staining of the palate shows normal squamous epithelium (black arrow), adjacent to the well-differentiated squamous cell carcinoma with parakeratosis, acute and chronic inflammation (blue arrow).

A biopsy of the palatal lesion was performed, which showed SCC (well-differentiated), with parakeratosis, acute, and chronic inflammation (Figure 3).

**Figure 4:**
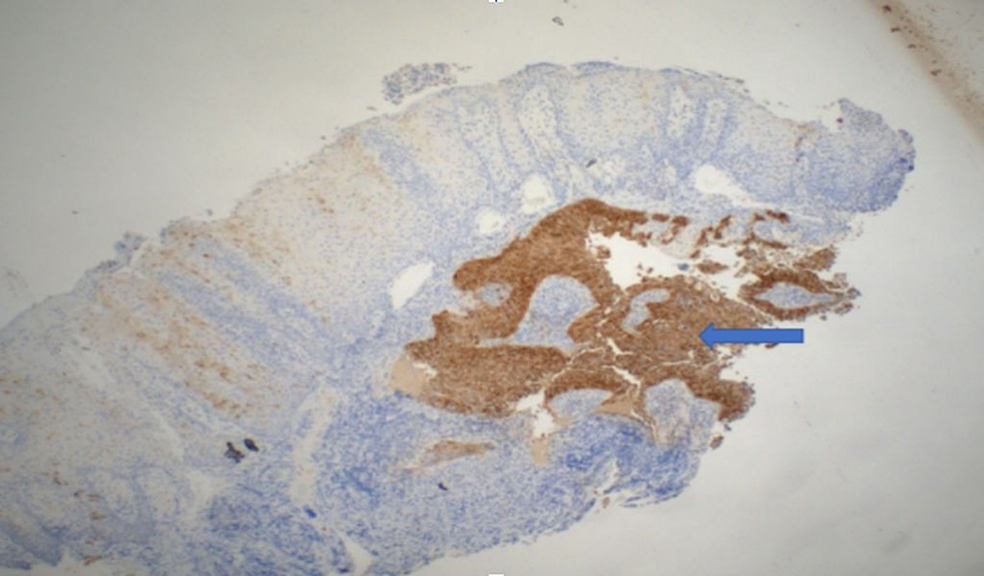
Immunohistochemistry by p16 shows diffuse nuclear and cytoplasmic staining of well-differentiated squamous cell carcinoma (blue arrow).

Immunohistochemistry by p16 shows diffuse nuclear and cytoplasmic staining of well-differentiated SCC (Figure 4). PD-L1 test showed a combined positive score of 10-15. CT chest, abdomen, and pelvis were negative for metastasis. NM bone scan showed radiotracer uptake in paranasal sinuses/facial bones. PET scan showed diffuse FDG activity in the hard palate, mildly hypermetabolic cervical, pretracheal, subcarinal, and left hilar nodes with minimal FDG activity. A diagnosis of locally advanced (stage IVa) oral cavity SCC was made. The patient was evaluated by a multidisciplinary team including an oncologist, head and neck surgeon, maxillofacial surgeon, radiation oncologist, and nutritionist for further management. The patient underwent prosthetic rehabilitation. A palatal obturator (prosthesis) was placed to improve his speech, eating, and prevent regurgitation. The patient opted for palliative care and did not want to pursue further treatment. He was discharged home.

## Discussion

Primary head and neck cancers are predominantly SCC. Over 90% of head and neck cancers are reported to be SCC [5]. There is a well-documented role of heavy tobacco and alcohol consumption to increase the risk of SCC by 5-25 folds. Infections including HPV, HIV, EBV, and chronic hepatitis C have also been associated with an increasing number of head and neck cancers, particularly oropharyngeal cancers [6,7]. SCC of the oral cavity can arise from premalignant lesions (erythroplakia, leukoplakia, and lichen planus) or rarely from normal mucosa. Initial presentation can be an ulcer (more likely in men) with necrotic border, nodular lesion, or exophytic mass [8]. The floor of the mouth and lateral or ventral surface of the tongue are the most common sites of oral OSCC. However, hard palatal perforation as an initial presentation of SCC has rarely been reported. Our patient had classic risk factors including extensive smoking history, alcohol consumption, and had chronic hepatitis c infection. Various histological variants of OSCC have been reported including well-differentiated (WD), moderately differentiated (MD), and poorly differentiated (PD). A study reported that men 41-60 years of age are more likely to develop oral SCC than females and 30.7% were reported to be well-differentiated OSCC type [9]. The staging system for HPV positive oropharyngeal cancer has been separated from HPV negative oropharyngeal cancer based upon a new International Collaboration on Oropharyngeal Cancer Network for Staging (ICON-S) system [10]. HPV positive OSCC has a better prognosis than HPV negative OSCC. Treatment of OSCC depends upon the stage of the OSCC. However, before initiation of the treatment pretreatment evaluation involving CT scan, MRI, and PET scan is mandatory to delineate the extent of the disease process. In addition, the site of the primary OSCC is also very important to determine the primary treatment modality. In stages, I and II of OSCC of hard palate surgery is the preferred treatment with an excellent outcome. Surgical intervention might involve resection of adjacent bony structures like palatectomy, alveolectomy, or maxillectomy (resection of the palatine process) [11]. Surgical defects can be reconstructed with mucosal flaps or a removable prosthetic (palatal obturator) can be utilized. Radiation therapy (RT) also offers optimal control of the primary lesion; however, the main limiting factor is radiation-related osteoradionecrosis of the maxilla. In advanced OSCC (stage III/IV), locoregional recurrence is frequent. Though data regarding the superiority of primary surgical intervention over definitive RT in hard palate OSCC are not well reported, surgical intervention is the preferred modality [12].

## Conclusions

This case highlights that OSCC of the hard palate can present as a palatal perforation. The diagnosis can be delayed especially in the geriatrics population with underlying cognitive impairment or dementia, where symptoms may remain unnoticed for a longer period. This case elucidates the patient-tailored approach to treat every patient in accordance with his/her wishes.
